# Supplementary material for: A high-resolution mRNA expression time course of embryonic development in zebrafish
Source: eLife. 2017 Nov 16;6:e30860. doi: 10.7554/eLife.30860 (PMC5690287; doi:10.7554/eLife.30860)
Supplement: Supplementary file 6. [file elife-30860-supp6.zip › biolayout-clusters-files/Cluster133-genes.html]

Cluster133


# Cluster133: Genes

| Ensembl ID | Gene Name | Chr | Start | End | Biotype |
| --- | --- | --- | --- | --- | --- |
| ENSDARG00000019338 | adprhl2 | 17 | 31594891 | 31602303 | protein\_coding |
| ENSDARG00000002790 | ap2m1a | 2 | 9991991 | 10020891 | protein\_coding |
| ENSDARG00000057882 | arpc3 | 21 | 16987366 | 16992828 | protein\_coding |
| ENSDARG00000041363 | dctn3 | 16 | 5245506 | 5254406 | protein\_coding |
| ENSDARG00000097057 | fxyd6l | 16 | 24686108 | 24727834 | protein\_coding |
| ENSDARG00000003375 | gng12a | 2 | 10503534 | 10555214 | protein\_coding |
| ENSDARG00000101844 | mibp2 | 18 | 5637596 | 5643151 | protein\_coding |
| ENSDARG00000104018 | mtpn | 4 | 895549 | 1054707 | protein\_coding |
| ENSDARG00000096118 | ost4 | 4 | 722947 | 724072 | protein\_coding |
| ENSDARG00000077934 | tegt | 11 | 198242 | 205794 | protein\_coding |
